# Localization of Radio Signal Sources for Situational Awareness Enhancement

**DOI:** 10.3390/s25237401

**Published:** 2025-12-04

**Authors:** Krzysztof Malon, Paweł Skokowski, Gregor Pavlin

**Affiliations:** 1 Faculty of Electronics, Institute of Communications Systems, Department of Mobile Communications Systems, Military University of Technology, 00-908 Warsaw, Poland; pawel.skokowski@wat.edu.pl; 2 Thales Nederland B.V., 2628 Delft, The Netherlands; gregor.pavlin@nl.thalesgroup.com

**Keywords:** situational awareness, cooperative sensing, communication, heterogeneous sensor network, localization

## Abstract

This article proposes a novel passive localization framework that leverages detection results from existing distributed radio detectors. The intuition behind this solution is to combine positive (signal detected) and negative (signal not detected) detection results with environmental data to refine localization estimates. Its novelty lies in providing a comprehensive, multi-dimensional framework for cooperative localization that enhances situational awareness by leveraging existing spectrum-monitoring capabilities. The proposed approach provides an additional functionality for a network of nodes monitoring spectral resources. It allows the transmitter’s location to be estimated based on the detection results of individual nodes. The unquestionable advantage of the proposed solution is that it does not require extra equipment or increased monitoring time. The developed method supports broad operational activities, e.g., tracking of authorized and unauthorized entities, and jammer localization. Using the proposed approach, one can increase efficiency in a given operational environment, and jammer localization. Using the proposed approach, one can increase efficiency in a given operational environment and situational awareness in a cognitive radio network. Furthermore, the experimental results of the estimation algorithm for an exemplary urban area indicate the legitimacy of a cooperative approach to the problem.

## 1. Introduction

The localization of radio transmitters is an essential element of modern security operations and directly impacts the level of situational awareness in a given area of operation. In a current operational environment, where information superiority is often crucial for tactical superiority, identification and localization of active sources of radio emissions allows for the detection and targeting of the unauthorized emissions (e.g., radios, communication systems, radars), analysis of the structure and location of control systems, counteraction against electromagnetic interference, planning of operational actions (e.g., precise strikes against unauthorized radio devices), and protection of own units by early detection of potential threats. The operation of localization systems in passive mode allows reconnaissance without revealing one’s position. Operational scenarios have changed significantly over the last few years. More and more security operations nowadays involve complex asymmetric environments [1]. Consequently, the sensing and communication requirements for these operations in urban terrain have also changed, emphasizing maximizing spectral and energy efficiency, for instance, in cooperative cognitive radio networks employing energy harvesting to prolong their operational lifetime [2]. This has driven the development of cooperative, communication-centric paradigms such as ISAC (Integrated Sensing and Communication), where existing communication signals, such as 5G NR (New Radio) OFDM (Orthogonal Frequency Division Multiplexing), are reused for device-free passive target localization, thereby enhancing spectral and energy efficiency [3]. Threats are harder to predict and can occur almost anywhere, which is a major challenge. It also means that areas must be persistently monitored by, e.g., Wireless Sensor Networks (WSN), often supported by UAVs (Unmanned Aerial Vehicles), to increase the probability of detection and coverage area. Such an architecture, particularly in Cognitive Radio (CR) networks, introduces significant challenges related to cooperation efficiency and communication overhead, which are addressed by developing advanced fusion rules that reduce the number of required sensing samples without degrading detection performance [4].

It is worth noting that the transmitter localization techniques developed and used in the security sector also apply in the civilian area, which fits into the concept of so-called dual-use. Such applications include the localization of emergency signals, the surveillance of the frequency spectrum in cities and industrial regions, and the detection of unauthorized emission sources, e.g., at airports or other critical infrastructure sites. In both security and civilian applications, the localization of radio transmitters enhances operational security and enables effective management of electromagnetic space.

The problem considered in this article concerns locating a radio signal transmitter. Radio transmitter localization can be classified according to various criteria, such as the data source, measurement technique, or system architecture. In general, two prominent cases related to this issue can be identified. The first one includes active systems, in which the transmitter cooperates with the localization system: it intentionally sends signals according to an established protocol, and receivers or measurement systems can use these signals for precise localization. In such a solution, time synchronization between devices is usually required, and high localization precision can be achieved by knowing the transmission parameters. However, this approach may not be possible or desirable in some applications. Therefore, passive systems that operate without the transmitter’s participation in the localization process have also been developed. In this case, the measurement system receives and analyzes the transmitted signals independently, without cooperating with the emission source. The receivers in such a system passively listen to the electromagnetic environment, ensuring discreet operation when locating unauthorized, hostile, or unknown transmitters. They can be used for signal intelligence (SIGINT), interference detection, or radio spectrum monitoring by regulatory services.

To conclude, it is worth emphasizing that the main motivation for addressing radio transmitter localization is its critical importance to modern security operations, as it directly affects situational awareness in the operational area. In the current operational environment, where information superiority is critical to tactical superiority, identifying, locating, and analyzing unauthorized radio emissions, countering interference, and planning offensive operations is essential. Scenarios involving complex urban environments, which require passive localization systems that enable reconnaissance without revealing one’s position, are particularly challenging. Moreover, advanced localization techniques have significant dual-use applications in the civilian sector, enhancing operational security and electromagnetic spectrum management, such as locating distress signals or detecting unauthorized emission sources. Despite the abundant literature on WSNs, there is a clear gap in a multidisciplinary approach to enhancing situational awareness in heterogeneous sensor networks that integrate various aspects, such as data fusion and limited node numbers in complex terrain.

### 1.1. Related Works

One of the most popular techniques for estimating the position of a mobile emitter is based on Measurements of Time Difference of Arrival (TDOA) and Frequency Difference of Arrival (FDOA) by multiple sensors [5,6]. The first method localizes by measuring the difference in the arrival times of electromagnetic waves at different sensors and converting it into distance. Suppose there is relative movement between the target and the sensors. In that case, measurements of the difference in frequency of arrival (FDOA) can be used to improve the accuracy of determining the target’s position and estimate its speed. Another well-known and widely used approach is the Angle of Arrival (AOA) method, which measures the direction of an incoming wave and determines the approximate location of the signal source by selecting the intersection point of the lines marking the directions of reception [7]. An example of an active system is a transmitter operating in a known, controlled environment that uses UWB (Ultra Wideband) technology. An example of the application of such a solution is the localization of an operator by the UGV (Unmanned Ground Vehicle) in the automatic following system [8]. It is also essential to consider conventional locating systems, such as GPS (Global Positioning System), which use the Time of Arrival (TOA) technique [9]. In this situation, the location is directly available by a radio signal source, and access to that information must be set by, for example, authorities or users. In GNSS (Global Navigation Satellite System) applications, especially in critical operations, it is necessary to consider the increasingly common threat of jamming or spoofing. As a result, positioning is impossible or falsified. Assisted GPS (A-GPS) has been developed to provide significantly improved capabilities, helping GPS work better and faster in almost any location, especially in buildings or when satellite signals are weak [10]. A-GPS provides the device with the necessary data via a radio network rather than the slow satellite link [11]. The need to localize user terminals has become a feature of subsequent generations of mobile cellular networks. The development has been from GSM (Global System for Mobile Communications) networks to 5G NR [12].

However, this paper focuses on the passive systems case where the localized transmitter does not cooperate with our system, such as the detection of illegally used transmitters in a given area by authorities controlling the proper use of spectrum resources or in the case of radio-electronic countermeasures (detection and localization of sources of unauthorized radio emissions). Another application of this localization method is determining the transmitter location for creating Radio Environment Maps (REMs) [13]. In this solution, the transmitter location and propagation models estimate the received signal level in the assumed region. Many methods use Received Signal Strength (RSS) to localize the transmitter [14]. These solutions achieve localization by exploring the relationship between the received signal strength and distance via wave attenuation. An example of such a solution could be a localization method supported by a Monte Carlo algorithm to improve accuracy [15]. Another issue in RSS-based methods relates to multiple coexisting sources and a complex propagation environment, especially the shadow effect [16]. Moreover, a Direction Of Arrival (DOA) localization method using convolutional neural networks is proposed to enable high-resolution localization of single or multiple radio frequency sources, even in challenging scenarios with low Signal-to-Noise Ratios (SNR) and closely spaced emitters [17]. A slightly different approach to localization was presented in the study [18], which examined the limitations of positioning accuracy using magnetic field data by comparing various fingerprinting algorithms. With the rapid development of UAVs, they have also found applications to increase the capabilities and efficiency of wireless sensor networks, including, among others, expanding the range of sensors by placing them on board the UAV, using the flying platform to collect data from sensor nodes (minimalization of sensor energy consumption) [19], and applying them in disaster management systems [20]. In the literature, one can also find works that focus on the fundamental task of locating radio transmitters, using the innovative Signal Doppler Frequency (SDF) method with the use of unmanned aerial vehicles as mobile receiving platforms, which is a distinguishing feature of the method compared to traditional techniques that require multiple stationary sensors [21,22]. Drone swarms are also used to detect and localize sources of electromagnetic emissions [23].

An essential issue in the literature is the location of sensors used in the spectrum-monitoring process, accounting for their parameters and propagation conditions [24]. Some works also focus on the optimization problem of selecting a subset of sensors from the deployed devices [25]. These techniques aim to optimize transmitter localization accuracy while constrained by limited resources. Although many studies on WSNs have been conducted [26,27], a multidisciplinary approach to enhancing situational awareness in heterogeneous sensor networks remains lacking. These studies strongly focus on specific sensor network aspects, e.g., data fusion algorithms [28], power efficiency [29], or a limited number of nodes [30] in homogeneous terrain.

### 1.2. Problem Definition

**Figure 1:**
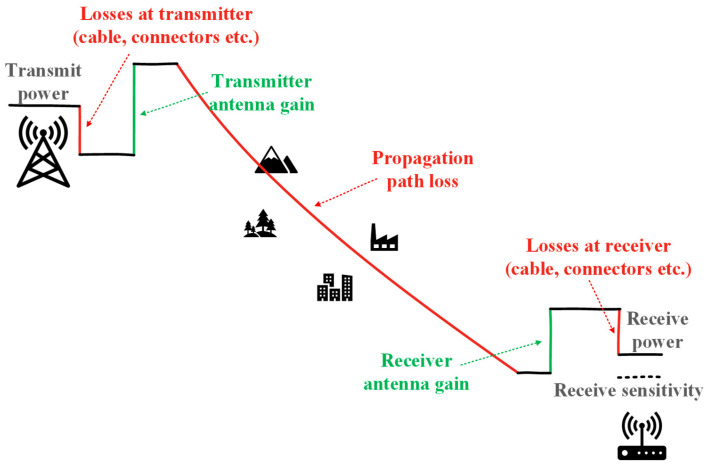
Radio link budget.

The proposed solution, which is described in detail in Section 2, is assumed to be an additional functionality for a network of nodes monitoring spectral resources, whose primary task is to detect radio signals in specific frequency bands. It integrates seamlessly with existing spectrum-monitoring networks, leveraging their primary detection capabilities to provide valuable location estimates without requiring new hardware or additional sensing effort, thereby extending the system’s overall utility. Based on the detection results from individual nodes and other data (described in detail in the next section), it is possible to estimate the transmitter’s location. It is worth emphasizing that no additional equipment or monitoring time is required to apply the proposed solution. The only effort required is to perform an additional calculation. The presented algorithm can also be used in a cognitive radio network, with spectrum-monitoring functionality as an additional element that enhances situational awareness. The considered method can extend the functionality of any distributed spectrum-monitoring system in both civilian and security domains. In summary, the proposed approach assumes the existence of a network of nodes performing the spectrum monitoring and detection process. On this basis, extending the existing system’s capabilities is possible by estimating the transmitter’s location. When operating several sensors within a network monitoring a given area and frequency range, it is also worth considering whether the results returned by individual sensors relate to the same transmitter. The presented algorithm uses radio signature/fingerprint methods for this purpose. Moving on to the issues linked to the proposed method, it should be noted that each sensor’s detection area depends on its location (propagation conditions), detector parameters (e.g., sensitivity), and the power level of the detected radio signal source. Those factors determine the radio link budget, i.e., received signal power in the detector (Figure 1).

**Figure 2:**
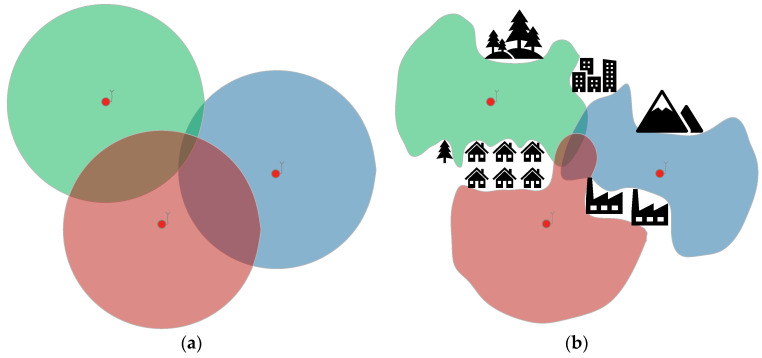
(**a**) Estimated radio signal source activity areas for an unknown location without additional data; (**b**) Estimated radio signal source activity areas for a known location with additional data. (The red dots means detectors.)

The foundational context of spectrum sensing in CR networks is comprehensively reviewed in [31], which provides essential background by classifying sensing techniques, detailing Energy Detection (ED), and emphasizing the role of Cooperative Spectrum Sensing (CSS) as a solution to critical challenges like fading, shadowing, and noise uncertainty. The core mechanisms of cooperative ED under complex propagation conditions are described in [32]. It presents a rigorous analytical framework for ED-based CSS over channels featuring multipath fading (Rayleigh) and shadowing (lognormal). This seminal work investigates data fusion (using techniques such as Square-Law Combining) and decision fusion (using the generalized k-out-of-n rule), deriving exact probabilities while explicitly accounting for reporting channel errors. Other operational challenges arise in practical scenarios, e.g., analyzing CSS using ED in the highly challenging low-SNR regime (0 dB to −25 dB) over Rayleigh fading channels. Ref. [33] presents a study that compares the performance metrics of various hard decision combining rules (OR, AND, L out of K) used at the Fusion Center (FC), demonstrating how spatial diversity enhances detection capability even under severe signal degradation. In addition, there are fusion methodologies involving different types of detectors, such as cooperative sensing in heterogeneous sensor networks, where nodes possess varied capabilities (e.g., combining ED statistics with eigenvalue-based detection statistics, such as Maximum Eigenvalue or Scaled Largest Eigenvalue) [34]. There are also advanced techniques for mitigating channel quality heterogeneity. In [35], the Authors examine CSS in fading environments and propose using optimization techniques to assign optimal weight vectors to cooperating sensors within a distributed detection and data fusion model. This approach enhances detection efficiency when nodes face non-uniform SNR values due to varying environmental factors and propagation effects. In general, results from multiple sensors (cooperative detection) increase detection efficiency [36]. Additional information on the location of individual detectors allows for estimating the area of the radio signal source. We can consider two cases for the analyzed sensor network: unknown or known operating areas. In the first case, it may be an ad hoc operation in an unknown area, without the option to obtain environmental data beforehand. To calculate the detection range based on the link budget, one can use statistical propagation models that estimate path loss for a given terrain type (e.g., free space, suburban, urban). However, they do not consider the specificity of a given area and terrain obstacles (buildings, forests, etc.). The designated areas are circles centered on each detector’s location, with a given radius (Figure 2a).

In a known area, a Digital Terrain Model (DTM) can be used. Knowing the site-specific elements, one can obtain a more accurate link budget that reflects realistic conditions. The estimated areas of radio signal source activity take irregular shapes, depending on the topography, and indicate transmitter locations much more precisely (Figure 2b). In the planning phase, it is also possible to analyze and select the detector locations. By fusing information from more sensors the uncertainty of the emitter whereabouts can often be significantly reduced. By using appropriate algorithms, detections of a source, as well as the lack of detections, can be used to improve estimation. The quality of the fusion depends to a great extent on the geographical distribution of radio detectors.

In this article, the authors do not specify the type of detector used but focus on the detector results and their potential for estimating the localization of radio signal sources.

### 1.3. Contributions and Paper’s Structure

The paper presents a cooperative approach to the fusion of radio detection data (both positive and negative). It proposes a framework for aggregating information from multiple sensors to reduce the uncertainty of the estimated transmitter location. This work’s novelty and practical significance lie in offering a comprehensive, multi-dimensional framework for cooperative localization of radio signal sources to improve situational awareness. The method uses data collected during real-life monitoring of the radio spectrum. After additional information is taken into account, the transmitter’s estimated location is determined. In this respect, it can be treated as an extension of the functionality of classical detection in electromagnetic space. This paper presents a method for collaborating with other systems, which can be used both to support the system’s work (to obtain additional information, such as transmitter power or antenna height, which is crucial for calculating the radio link budget) and to upload the results of its work to a data fusion system (can be part of a more complex system of heterogeneous sensors, each of which brings different added value). Unlike other works focusing on specific measurement techniques or challenges, this paper presents a novel methodology for combining and processing information (even binary) from various radio detection sensors and environmental data, to estimate the emitter’s whereabouts with better precision, thus increasing the operational efficiency.

The main contributions of this paper are summarized below:

- Analysis of the current state of radio transmitter detection, resulting in the concept of a method enabling the expansion of functionality to include determining the transmitter’s location zones.
- Implementation of a flexible, passive approach to determining transmitter location in a simulation environment.
- Detailed research and analysis of results for the defined requirements and research scenario, demonstrating the benefits of using the proposed approach. Identification and confirmation of the significance of detection data indicating the absence of a signal in estimating the signal source’s location.
- Demonstration of the possibility of combining the proposed approach with other data (e.g., context and terrain data) and systems to achieve synergy between solutions.

Table 1 compares our proposed cooperative localization framework with conventional approaches, highlighting the strategic advantages enabled by its multifaceted design. The architecture integrates passive operation with existing spectrum-monitoring functionality, enabling accurate localization without the synchronization costs inherent in methods such as TDOA/FDOA or the need for dedicated infrastructure. Furthermore, the framework establishes a robust, energy-efficient solution for refining transmitter location zones by fusing simple binary detection results with multidimensional environmental data, such as Digital Terrain Elevation Data (DTED).

The rest of this paper is organized as follows. Section 2 presents the proposed solution, including a detailed description of the authors’ method. Section 3 contains the research part of the considered solution, including assumptions, research scenario, and discussion of the results. Finally, concluding remarks are outlined in Section 4.

## 2. Proposed Solution

**Figure 3:**
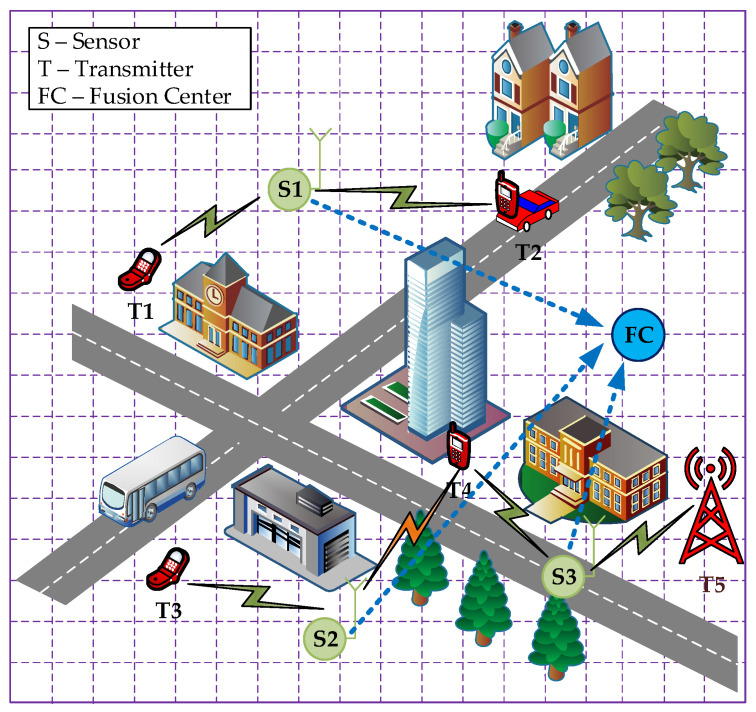
Example illustration of the proposed system application.

What seems to be lacking is a multidisciplinary approach to situation awareness enhancement based on a heterogeneous sensor network. In that case, knowledge more effectively reflects reality. Dedicated sensors can be used, whose task is only to conduct spectrum monitoring, or platforms that use cognitive radio technology, which transmit with other users and perform sensing to improve electromagnetic awareness [37,38,39]. From an operational point of view, the more complex and dynamic the environment, the more relevant information is needed. Thus, in complex scenarios, deploying many sensors and pervasive computing must be complemented by an adaptive sensing and fusion capability to extract relevant information for mission success. The basic information obtained in the radio spectrum-monitoring process is awareness of radio emissions in the specified bands. Many radioactivity detectors exist, e.g., energy, feature-based, cyclostationarity, etc. The detection result is information about a radio signal activity at a given frequency. When the detector cooperates with the classifier, it can also determine the transmission type for identification purposes (authorized or unauthorized entities). The proposed algorithm further contributes to situational awareness by indicating the estimated position of the radio transmitter (signal source). This solution demonstrates how the localization of a radio signal source (transmitter) using radio detectors (sensors) can be used for situational recognition. Figure 3 presents an example illustration of the proposed system application. Sensors (S1, S2, S3) represent a distributed radio detector network that passively monitors spectral resources. Each sensor in the network performs a detection process and generates a simple binary result: either positive if a signal is detected, or negative if no signal is detected. Transmitters (T1–T5) are radio signal sources that the system aims to locate. The arrows in the diagram, pointing from transmitters to sensors, represent the detection relationship. An arrow indicates that a given transmitter is within the detection range of a specific sensor, which depends on factors like transmitter power, sensor sensitivity, and propagation conditions. Conversely, the absence of an arrow signifies that the transmitter cannot be detected by that sensor (e.g., outside its coverage area). Fusion Center (FC) is the central element of data aggregation and fusion. As indicated by the arrows from the sensors to the Fusion Center, the FC receives the binary sensing results from all individual sensors in the network. The core algorithm is executed here to process the collected data to determine the transmitter’s location.

**Figure 4:**
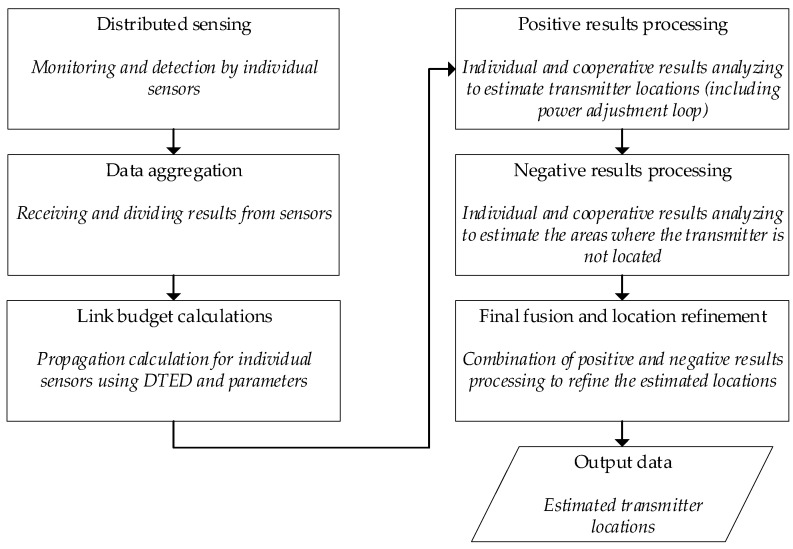
Top-level functional block diagram of the proposed cooperative passive localization system.

To provide a comprehensive overview of the proposed methodology, Figure 4 illustrates the top-level functional architecture of the cooperative passive localization system. This framework visualizes the sequence of data-processing and fusion steps used to leverage distributed radio-detection results for improved situational awareness. The process begins with distributed sensing, in which individual sensors monitor the spectrum and produce simple binary results indicating the presence or absence of a signal. These outcomes are forwarded to the Fusion Center, where they are received and split into positive and negative sets (data aggregation). Crucially, the methodology incorporates simultaneous link budget calculations. This step uses inputs such as Digital Terrain Elevation Data and sensor and transmitter parameters (e.g., power and antenna height) to model the theoretical detection ranges for each sensor. The core localization procedure is divided into the following functional blocks:

- Positive result processing: analyzes detections to estimate potential location areas; this stage is designed with a power adjustment loop to resolve scenarios where individual sensor coverage areas initially fail to overlap, suggesting an underestimated transmitter power assumption,
- Negative result processing: analyzes non-detections to estimate areas where the transmitter is confirmed not to be located,
- Final fusion and location refinement: combines positive and negative processing results to refine the estimated location.

This integrated approach yields the output data—the estimated transmitter locations.

The proposed solution assumes that the analyzed area, as depicted in Figure 3, is divided into discrete subsets of a specific size and represented by a matrix containing geographic coordinates for each bin:

(1) $$C=\left[\begin{array}{ccc} c_{11} & \ldots & c_{1n}\\ \vdots & \ddots & \vdots \\ c_{m1} & \ldots & c_{mn} \end{array}\right],$$

where

- m, n—indices of the matrix C that define its size,
- $c_{mn}$—geographical coordinates of bins.

The number of all bins is equal to the number of elements of the matrix C:

(2) $$N_{c}=\left|C\right|=m\cdot n,$$

$N_{s}$ sensors are placed in the analyzed area, and each sensor (depending on its location and parameters, and also taking into account propagation conditions determined by the terrain profile as well as obstruction/shadowing effects) can detect the radio signal generated by transmitters located in specific ranges. For a given sensor s, a matrix $D^{\left(s\right)}\;$of the same dimensions as matrix C can be determined, containing the values of:

- 0—indicating the inability to detect a signal generated by a transmitter located in a specified bin;
- 1—indicating the possibility of detecting such a signal:

(3) $$D^{\left(s\right)}\in {\left\{0,1\right\}}^{m\times n},$$

where

- s—sensor index,
-
$s\in \left\{1,\ldots ,N_{s}\right\},$

- $N_{s}$—number of sensors placed in a given area.

Considering a given bin, let introduce $\left(i,j\right)$ representing its indices in the analyzed matrix $D^{\left(s\right)}$. Depending on the number of sensors used, their locations and parameters, and the power of the localized transmitter, a given bin may be monitored by more than one sensor. In such a case, an opportunity arises to leverage this fact when analyzing the results. Let us define a matrix containing values for each bin, denoting the number of sensors that cover a given part of the area with their detection range:

(4) $$O\in {\left\{0,1,\ldots ,N_{s}\right\}}^{m\times n}.$$

Let us also introduce the notation of o for the variable representing the individual elements in the above matrix.

To facilitate further study, we now switch from matrix notation to set notation, denoting each matrix element by the corresponding index pair in a set. From the perspective of subsequent analysis, the intervals for which these sensor matrices $D^{\left(s\right)}$ contain the value 1 are of particular importance. For each sensor, let define a set containing the indices $\left(i,j\right)$ of such bins:

(5) $$R^{\left(s\right)}=\left\{\left(i,j\right)|D_{i,j}^{\left(s\right)}=1\right\}.$$

The content of the above set for each sensor s in conjunction with the matrix C defines a certain geographic area composed of discrete fragments, and the size of such a set reflects the area of the terrain (given the known size of a single bin). Assuming that each sensor performs the process of monitoring and detection of the radio environment, in the simplest approach it returns one of two possible results: s—detection (presence) of a signal within the monitored range (positive result), or $M^{\left(s\right)}=0$—no signal (negative result). Depending on the $M^{\left(s\right)}\;$ results, $R^{\left(s\right)}$ sets will also be used differently in subsequent processing steps. Let us denote as $R_{p}^{\left(s\right)}$ the set of bins indices, determined in the case of a positive result of sensor s detection ($M^{\left(s\right)}=1$), in which the transmitter is potentially located. Alternatively, in a situation where sensor s returns a negative detection result ($M^{\left(s\right)}=0$), the $R_{n}^{\left(s\right)}$ set will denote the area in which the presence of the transmitter is not estimated. Therefore, let us denote by $N_{s,p}$—the number of sensors reporting a positive result, while by $N_{s,n}$—the number of sensors reporting a negative result, and then the equality occurs:

(6) $$N_{s}=N_{s,p}+N_{s,n}.$$

In the subsequent processing steps, resulting in the determination of the estimated transmitter localization, it is assumed that at least one sensor returns a positive result ($N_{s,p}>0$). Suppose none of the sensors report a positive result ($N_{s,p}=0$). In that case, it means there is no transmitter in the analyzed area, or that it is located outside the sensors’ detection range. If the detections can be associated with an identified transmitter (e.g., using radio fingerprint recognition), then results $R_{p}^{\left(s\right)}\;$from individual sensors can be fused. To ensure that the different sensors provide information about the same transmitter, it is also essential to synchronize the timing of sensing operations (or timestamp each result) and to frequency-match (monitor on the same frequency bands).

At this stage, having the determined sets for individual sensors, it is already possible to determine the potential areas of transmitter location ($R_{p}^{\left(s\right)}$) and zones where it does not occur ($R_{n}^{\left(s\right)}$). The next steps can then proceed to fuse these results to more precisely determine the location of the transmitter. The fusion of information from several sensors reporting the detection is carried out by finding the intersection of $R_{p}^{\left(s\right)}$:

(7) $$R_{p}=\overset{N_{s,p}}{\underset{s=1}{⋂}}R_{p}^{\left(s\right)}.$$

The $R_{p}$ set is the common part of the individual $R_{p}^{\left(s\right)}$ sets. On the other hand, to determine the area where the transmitter is not present, ${the\;union\;of\;R}_{n}^{\left(s\right)}\;is\;required$:

(8) $$R_{n}=\overset{N_{s,n}}{\underset{s=1}{⋃}}R_{n}^{\left(s\right)}.$$

Note that no identification of sources is required to compute $R_{n}.\;\;$Using the above aggregated information, one can finally determine a set that contains the indices of bins in which the position of the transmitter is estimated:

(9) $$R_{p,n}=R_{p}\backslash R_{n}.$$

In order to provide the size of the designated area, the size of this set (number of bins) can be calculated:

(10) $$N_{r}=\left|R_{p,n}\right|.$$

Algorithm 1 presents the method for estimating the area of radio signal source activity in pseudo-code. The algorithm includes successive processing steps to analyze results for the same transmitter, but in general, the proposal is designed to handle multiple transmitters. The procedure for estimating the location of each signal source is analogous to the single case presented below. In such cases, additional functionality, such as a radio signature/fingerprint [40,41], is needed to distinguish between transmitters.
**Algorithm 1** *(Estimating the area of radio signal source activity)***Input:** sensing results from individual sensors ($M^{\left(s\right)}$), sensors locations, sensors parameters (sensitivity, antenna height), transmitter parameters (power, antenna height), and Digital Terrain Elevation Data.
1.Divide sensor results into two groups:#1—contains indexes of sensors that detected the presence of the signal ($M^{\left(s\right)}=1$),#2—contains indexes of sensors that did not detect the presence of the signal ($M^{\left(s\right)}=0$).2.Get the first set #1.3.Estimate transmitter locations $R_{p}^{\left(s\right)}$ based on positive results from individual sensors.4.Estimate transmitter locations based on all positive results—intersection of the $R_{p}^{\left(s\right)}$ sets ($R_{p}=\overset{N_{s,p}}{\underset{s=1}{⋂}}R_{p}^{\left(s\right)}$).**if** the estimated transmitter area is empty ($R_{p}=\emptyset$) **then**
   5.Increase transmitter power $P_{T}$.    6.Back to point 3.**end if**7.Get the second set #2.8.Estimate the areas $R_{n}^{\left(s\right)}$ in which the transmitter is not located based on negative results from individual sensors.9.Estimate the area in which the transmitter is not located based on all negative results—the sum of the $R_{n}^{\left(s\right)}$ sets ($R_{n}=\overset{N_{s,n}}{\underset{s=1}{⋃}}R_{n}^{\left(s\right)}$).10.Estimate transmitter locations based on all available (positive and negative) results—difference between sets $R_{p}\;$ and $R_{n}$ ($R_{p,n}=R_{p}\backslash R_{n}$).11.**Output:** estimated transmitter locations $R_{p,n}$.


The required inputs for the algorithm are: sensing results from individual sensors, indicating either the presence ($M^{\left(s\right)}=1$) or absence ($M^{\left(s\right)}=0$) of a signal; the geographical locations of the sensors; sensor parameters, such as sensitivity and antenna height; transmitter parameters, like power and antenna height, which can be obtained from other systems or contextual information; Digital Terrain Elevation Data to account for propagation conditions. In the first step, the results are segregated. The data from spectrum monitoring carried out by individual sensors is divided into two subsets. The first is a set of results that indicate the presence of a radio signal ($M^{\left(s\right)}=1$), while in the second one, the results of the absence of this signal are stored ($M^{\left(s\right)}=0$). Then processing positive detections begins by estimating the potential location area, denoted as $R_{p}^{\left(s\right)}$, for each sensor that reported a positive result. This area represents all possible locations from which a transmitter could have been detected by that specific sensor, based on the radio link budget. To perform this process, it is necessary to provide information on the transmitter power, sensor parameters (location, sensitivity, and antenna height), and the terrain elevation model, including buildings, if available. At this point, it is worth emphasizing that the proposed solution can interact with other systems. For example, the transmitter power and antenna height (required for the calculation) can be determined based on different data sources. In such a situation, one can use contextual information, for example, on transmitters operating in the analyzed frequency band, transmitters located in a given area (reconnaissance information), or unauthorized equipment (e.g., jamming stations). As part of this stage, it is necessary to perform calculations to determine the path loss values on individual links. In this case, one can use ready-made solutions, e.g., HTZ Warfare [42], or develop one, as in the article [43]. The more faithfully the real conditions are reflected, the more accurate the determined location. Next, the algorithm fuses the information from all positive results by calculating the intersection of all individual $R_{p}^{\left(s\right)}$ sets. According to all sensors that detected it, the transmitter is potentially located in this common area. Within the 4th point of the algorithm, the power adjustment loop is executed if needed. The algorithm checks if the resulting intersection area is empty. An empty set indicates that the estimated areas from individual sensors do not overlap, which could be due to an incorrect (underestimated) transmitter power assumption. In that case, the assumed transmitter power must be increased, and the process must be repeated from the previous step to recalculate the intersection. The algorithm then processes the group of sensors with negative results. For each of these sensors, it estimates the area $R_{n}^{\left(s\right)}$ where the transmitter is not located. This is the area within the sensor’s theoretical detection range where the signal was not found. In the following step, the algorithm aggregates all negative information by calculating the union of the individual $R_{n}^{\left(s\right)}$ sets. This combined area represents all locations where the transmitter is confirmed to be absent, based on negative detections. The final and most crucial step is refining the location estimate by combining positive and negative results. This is done by taking the set difference between the area derived from positive detections ($R_{p}$) and the area excluded by negative detections ($R_{n}$). The algorithm’s output is the final set $R_{p,n}$, which represents the estimated geographical locations of the radio signal source. This entire procedure is designed to be repeated for different transmitters, which can be distinguished using methods like radio fingerprinting.

The results of the radio localization process can be further enhanced by considering the context and the knowledge about the areas where the transmitter can/cannot be positioned. For example, certain areas are not accessible to the platforms typically carrying the transmission equipment due to deep water, swamps, dense forests, steep slopes, or heavily defended areas. Inherently uncertain information about such areas can be represented using probabilistic context maps, which can be fused with the outputs of the radio localization algorithm using Context-boosted Particle filters [44]. This is a straightforward Bayesian fusion method that can efficiently handle uncertain context knowledge about mobility and the complex geometric shapes of the possible transmitter locations estimated by the presented algorithm. Moreover, this approach is suitable for fusing radio localization outputs with other sensors observing dynamic targets and for assessing the target’s whereabouts in surveillance gaps, an essential ability for tracking unauthorized entities [45].

## 3. Simulation Studies and Results

The proposed solution presented in Section 2 has been implemented in MATLAB 2024b. This part contains the assumptions, research scenario, results, interpretation, and discussion.

### 3.1. Assumptions and Scenario

**Figure 5:**
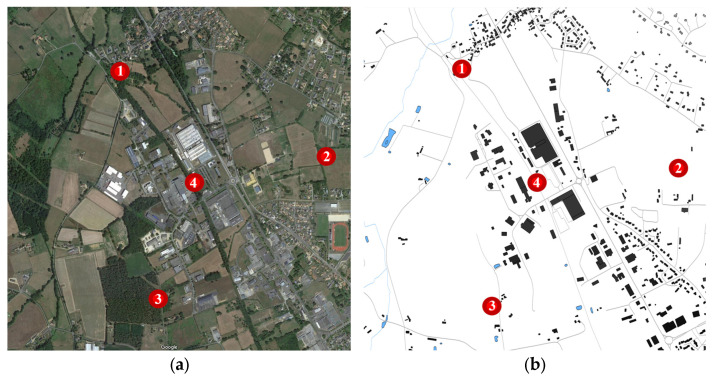
(**a**) Analyzed area with sensor locations on Google Maps; (**b**) Analyzed area with sensor locations on the map with terrain data for path loss calculations. (The red dots means sensors).

The 2 km × 2 km urban area was selected for the tests, with four sensors placed at arbitrary locations (Figure 5).

The area shown in Figure 5 was discretized into 1681 cells (i.e. bins), each measuring 50x50 meters. In the experiments, the transmitter was systematically moved through the 1681 locations, each corresponding to the center of a cell. For each bin, potential transmitter locations were estimated according to the presented method. The radio environment module calculated path-loss values between radio transmitters and sensors [46]. During the computations, this module considers the digital terrain model and shadowing effects introduced by buildings. The received signal level at the sensor input, which enables correct signal detection, was set to −110 dBm. Radio link budget calculations (i.e., path loss, sensor sensitivity, and assumed transmitter power) were performed for each potential transmitter location to determine each sensor’s coverage area. According to the proposed algorithm, combining results from multiple sensing devices enables more precise estimation of the transmitter location. A coverage factor (CF) measure was introduced to quantify the size of the estimated area of the radio transmitter location:

(11) $$CF=\frac{N_{r}}{N_{c}}\cdot 100\%,$$

where

- $CF\in \left[0\%,100\%\right]$.

This metric shows the percentage of the calculated radio signal activity region relative to the whole area. The lower the value of the coverage factor, the more precise is the estimate of the location of the signal emitter.

### 3.2. Results and Discussion

**Figure 6:**
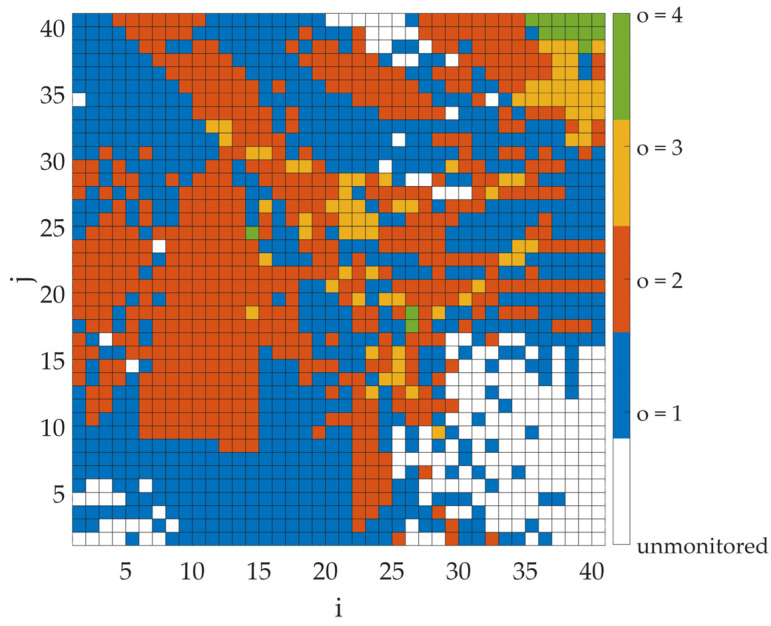
Coverage map for cooperating sensors (O matrix) for a transmitter power of −10 dBm.

This section presents the simulation results of the proposed algorithm based on the detection results from the four sensors presented in the scenario. Figure 6 shows the coverage map for cooperating sensors, presented as a graphical representation of the O matrix for a fixed transmitter power level. Note, the figure does not show the estimated whereabouts of the transmitter. Instead, the color of each bin corresponds to the number of sensors that would cover the emitter if it were in that bin. As mentioned earlier, there may be bins in the analyzed area that are outside the sensors’ detection range (marked in white). The designation of such zones is also a significant outcome, especially from a security operations perspective. Thanks to such an analysis, additional sensors can be deployed to cover a hitherto unmonitored area or to provide physical protection for specific zones. In other cases, individual colors indicate the number of sensors (o) covering a given bin.

**Figure 7:**
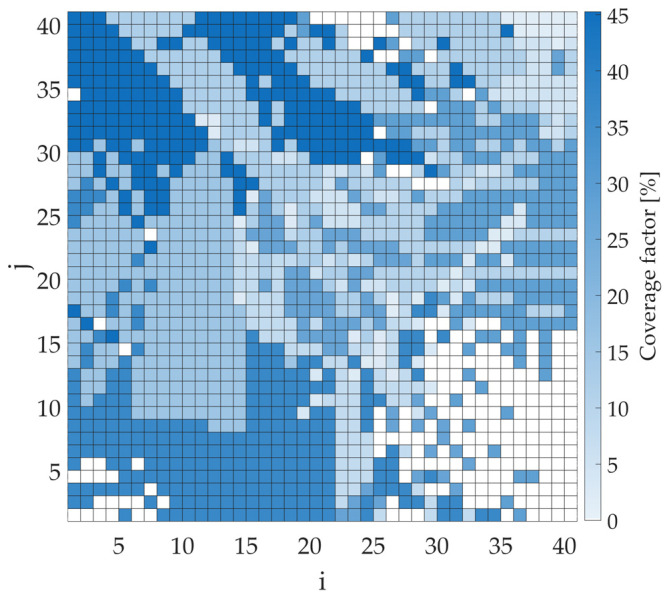
Coverage factor map for cooperating sensors considering only positive ($M^{\left(s\right)}=1$) results for a transmitter power of −10 dBm.

**Figure 8:**
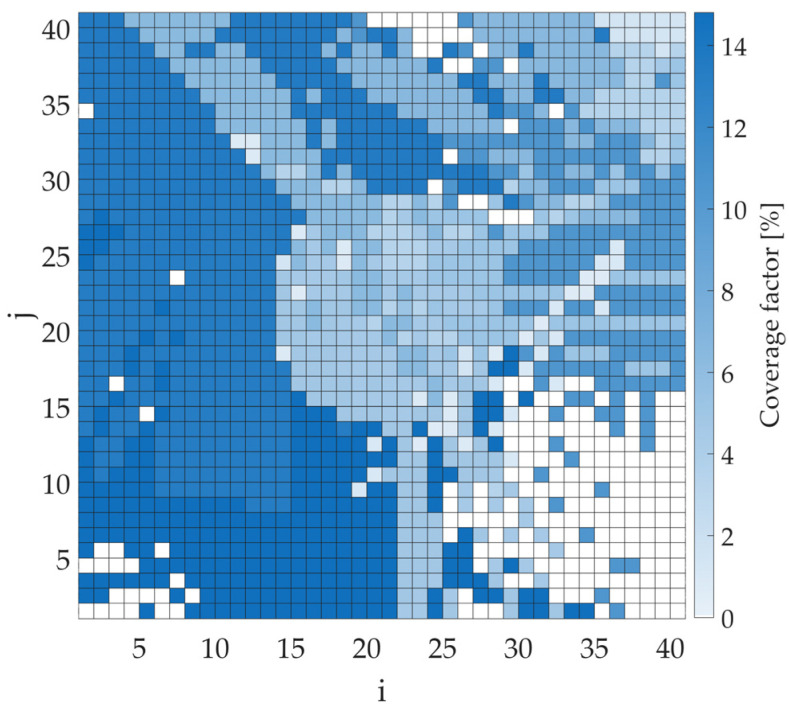
Coverage factor map for cooperating sensors considering both positive ($M^{\left(s\right)}=1$) and negative ($M^{\left(s\right)}=0$) results for a transmitter power of −10 dBm.

In the next step, the coverage factor values were calculated assuming the transmitter appears in each bin, with full cooperation from all four sensors. These simulations were carried out in two steps to observe the impact of considering the different types of results. In the first one, only the positive results returned by the sensors were analyzed ($M^{\left(s\right)}=1$). On the other hand, in the second case, both positive ($M^{\left(s\right)}=1$) and negative ($M^{\left(s\right)}=0$) were taken into account. The results of the coverage factor values in graphical form are shown in Figure 7 and Figure 8 for the first and second cases, respectively. Note, the figures do not show the estimated whereabouts of the transmitter. Instead, the color of each bin corresponds to the CF that would be obtained if the emitter were located in that bin. A coverage factor of zero indicates bins outside the sensors’ detection range. The importance of considering both types of results is evident in comparing, e.g., the maximum CF values for both cases. When only positive values are considered, the maximum CF value is 45.27%. This rate can be significantly reduced when negative results are also considered. Then the maximum CF value decreases to 14.81%, significantly more accurately estimating the transmitter’s position.

**Figure 9:**
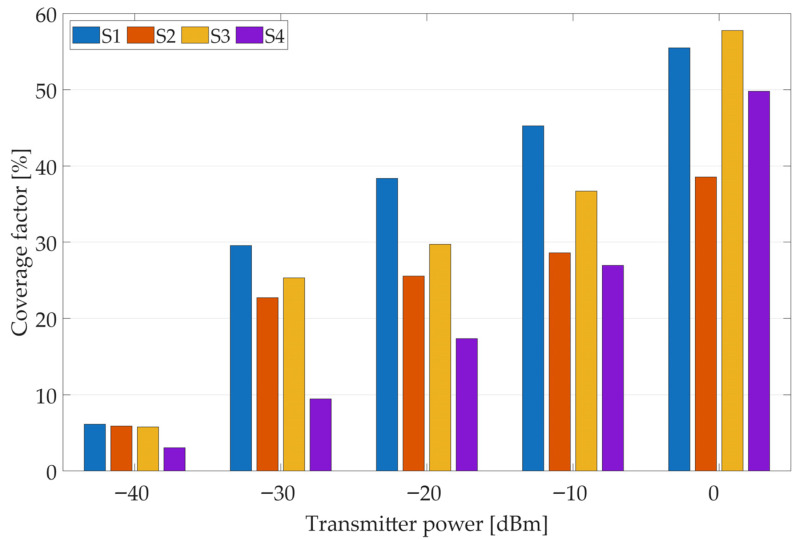
Coverage factor values for individual sensors considering only positive ($M^{\left(s\right)}=1$) results for different transmitter power levels.

**Figure 10:**
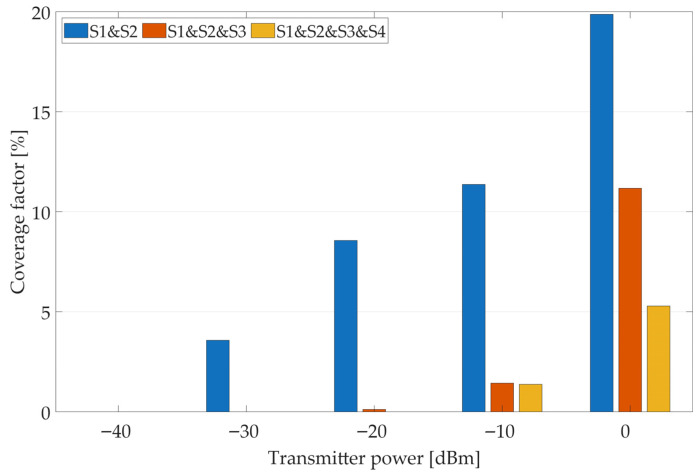
Coverage factor values for cooperating sensors considering only positive ($M^{\left(s\right)}=1$) results for different transmitter power levels.

Figure 9 and Figure 10 show the coverage factor calculations for different transmitter powers and sensor configurations (individual sensors and the cooperation of several sensors, where S1 means sensor no. 1, S2—sensor no. 2, S3—sensor no. 3, and S4—sensor no. 4). In this case, due to the large number of possible variants, the analysis focuses on the scenario in which the algorithm considers only positive outcomes (i.e., signal detections). As shown earlier, additional information about negative results (i.e., the absence of signal detection by a given sensor) can also be incorporated, significantly improving transmitter localization accuracy. In each sensor configuration, as the transmitter’s power increases, the coverage factor also increases, i.e., the area of potential transmitter locations expands. Analyzing results from individual sensors, it can be seen that the estimated area size depends on the sensor’s location; e.g., sensor no. 4, with low transmitter power levels, has smaller coverage factor values than the other three sensors due to its location between buildings. On the other hand, if a transmitter is within its coverage area, determining its location will be more accurate. When information from an increasing number of sensors is considered, a more precise estimate of the transmitter’s position can be obtained (with a smaller coverage factor). The presented results illustrate the importance of determining transmitter power (based on contextual information from other sources, as mentioned earlier).

**Figure 11:**
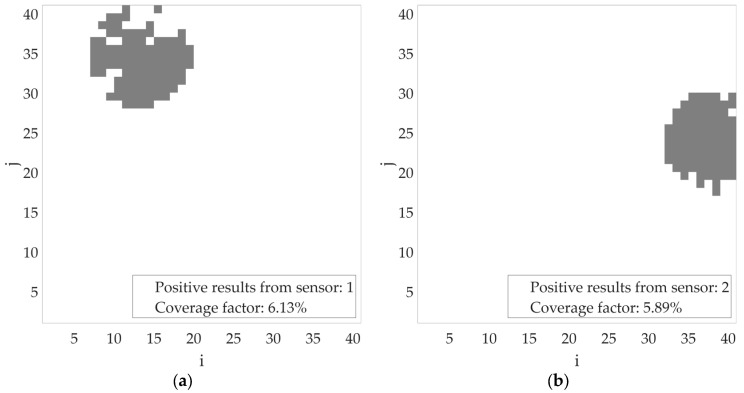
Estimated area of radio signal sources activity for transmitter power = −40 dBm (**a**) based on sensor no. 1 results; (**b**) based on sensor no. 2 results.

**Figure 12:**
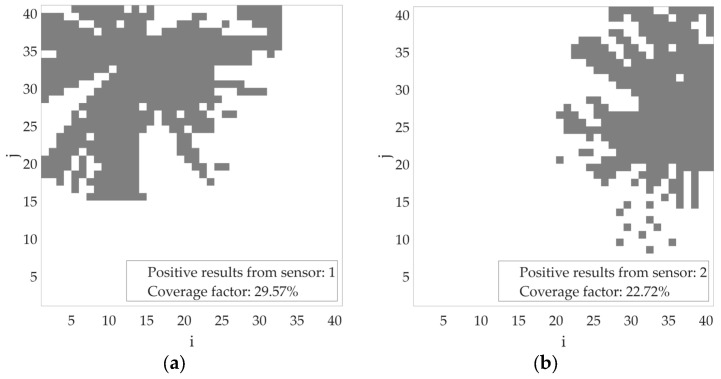

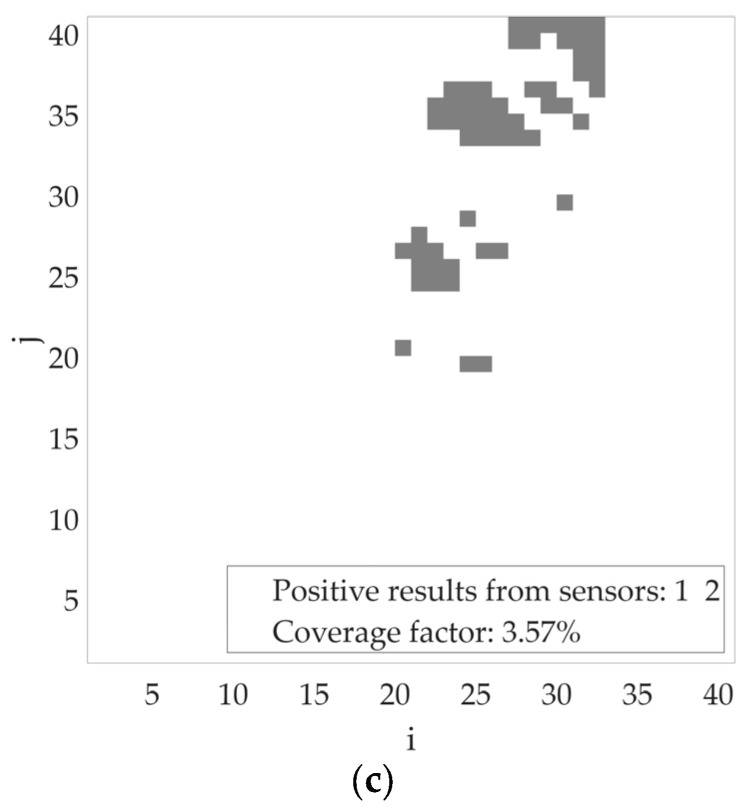
Estimated area of radio signal sources activity for transmitter power = −30 dBm (**a**) based on sensor no. 1 results; (**b**) based on sensor no. 2 results; (**c**) based on sensors no. 1 and 2 results.

Based on the simulations performed, let us consider an example scenario of the proposed algorithm’s operation for the incorrect determination of transmitter power (missing or incomplete contextual information). In the figures below, the bins where the transmitter position is estimated are marked in gray. Let us assume an initial transmitter power of −40 dBm. If, during operation, both sensor no. 1 and sensor no. 2 return a positive detection result, the transmitter power should be increased, as the areas estimated by the individual sensors do not overlap (Figure 11). Such a variant has been accounted for in the proposed algorithm. After changing the assumed transmitter power level to, for example, −30 dBm, the estimated area (marked in gray) of the transmitter location for individual sensors increased, as shown in Figure 12.

**Figure 13:**
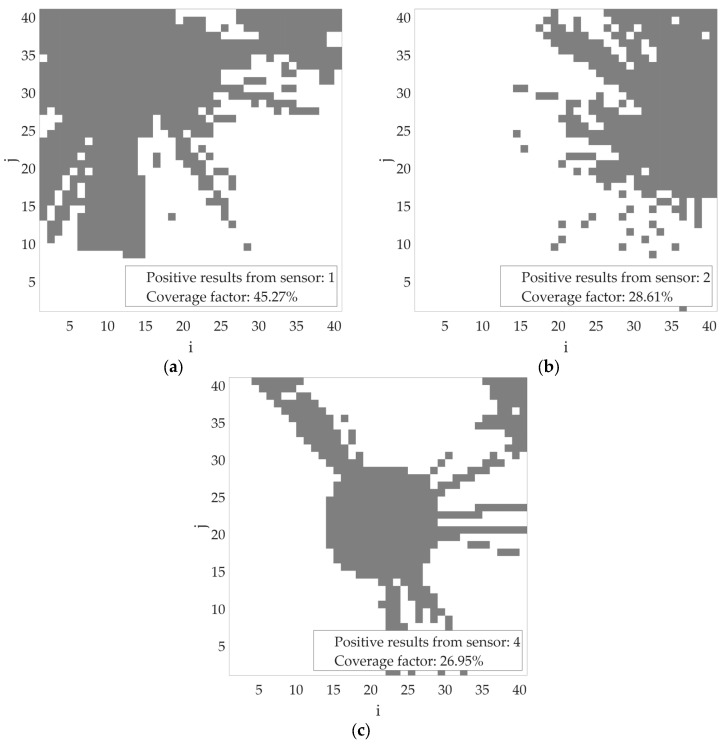
Estimated area of radio signal sources activity for transmitter power = −10 dBm (**a**) based on sensor no. 1 results; (**b**) based on sensor no. 2 results; (**c**) based on sensor no. 4 results.

**Figure 14:**
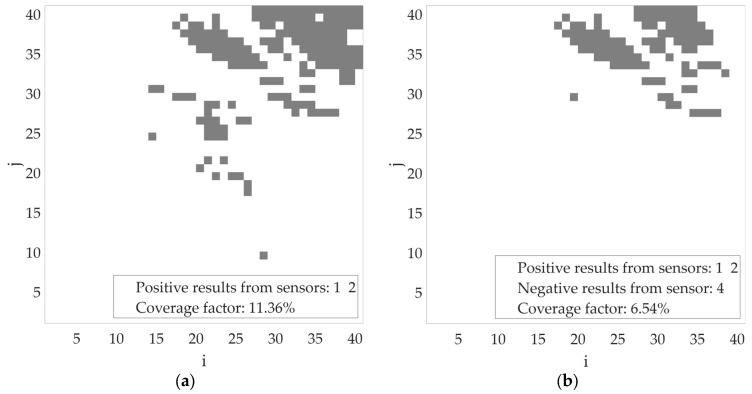
Estimated area of radio signal sources activity for transmitter power = −10 dBm (**a**) based on sensor no. 1 and 2 results; (**b**) based on sensor no. 1, 2, and 4 results.

Considering the results from both sensors, the coverage factor is about 3.5% (Figure 12c)—further increases in transmitter power result in a larger estimated transmitter location area. To illustrate the gain from using the proposed algorithm with several sensors, additional tests were conducted with the transmitter power set to −10 dBm. Firstly, Figure 13 shows the estimated coverage areas of individual sensors. Figure 14a presents the aggregated outcome based on the results from sensors no. 1 and 2, assuming that both detected a radio signal from the same transmitter. As shown, the designated area (coverage factor = 11.36%) has been significantly reduced. In the presented scenario, it was assumed that sensor no. 3 did not return any, while sensor no. 4 returned a result without a radio signal (negative result). Based on this result, the estimated location of the radio transmitter is further constrained (coverage factor = 6.54%), as shown in Figure 14b.

## 4. Conclusions

This article presents the concept of using radio detectors that cooperate to monitor radio signal sources and estimate their locations. Using the proposed approach, one can improve situational awareness efficiency. The results confirm that the cooperative approach significantly improves situational awareness quality. An indisputable advantage of the proposed solution is its ability to operate without additional costs—i.e., without additional sensing—by leveraging information from the detection process already carried out. A proposed method for localizing radio signal sources to enhance situational awareness in a multi-context approach might be integrated with, e.g., radio direction finders as a first step in the localization procedure. Additionally, in the security context, in combination with image recognition, it can significantly reduce the search area for threats and speed up operational activities. Moreover, during the mission planning phase, the proposed solution, combined with map information, can also be used to determine whether sensors cover an area of interest or indicate potential locations of the radio signal source, while accounting for building infrastructure. Another element of the multi-context approach is the use of transmitter power information from various data sources and radio signature methods to determine whether results reported by individual sensors are related to the same transmitter. Furthermore, thanks to a heterogeneous sensor network and the combination of results from different types of detectors, it is possible to build a complete situational awareness that supports various functionalities, such as asset tracking and even jammer localization. The authors plan to continue researching additional sensors and to combine this information with the results of image recognition and radio fingerprint solutions. Finally, the signal strength received by individual sensors can also be considered to estimate the transmitter’s potential location even more accurately.

## Figures and Tables

**Table 1:** Comparison of the proposed solution with existing approaches.

Novelty/Feature	Existing Approaches	Goal	Remarks
Passive operation & architecture	TDOA/FDOA requires strict time/frequency synchronization; UWB requires specialized active infrastructure/anchors; GNSS relies on external satellite signals	Low resource consumption and cost, stealthy operation, and utilization of existing sensing platforms	The proposed approach is inherently passive, leveraging data from existing Spectrum Monitoring/Cognitive Radio sensors; it avoids the high overhead and synchronization costs
Extension of existing functionality	Dedicated localization systems, e.g., SDF implemented on moving UAVs, dedicated professional TDOA/FDOA systems	Provide localization capability as an integrated feature of existing cognitive radio/spectrum-monitoring networks for situational awareness enhancement	Our method is an additional functionality for networks already performing spectrum sensing, such as those relying on energy detection; it is not intended to compete directly with high-precision (but often resource-intensive) methods like TDOA/FDOA or UWB, but rather to complement them by providing estimated location zones based on binary data where other methods may be unavailable or too costly to deploy
Fusion of binary (positive/negative) detection results	Classical CSS focuses on maximizing detection probability for spectrum access; RSS-based localization relies on raw signal strength; SDF is based on complex geometric processing of Doppler shifts	Enhanced cooperative efficiency and robustness to noise uncertainty with minimal signaling overhead	The proposed solution uses simple binary results (the fusion of both positive and negative detection results) with minimal data transfer, contributing to energy efficiency; this is complementary to (rather than competing directly with) high-precision systems
Multi-dimensional fusion leveraging real environmental/context data	Traditional RSS-based ranging assumes simple path loss models; SDF relies on moving sensors and specialized Doppler processing	Robustness and accuracy in complex urban/NLOS environments by precisely defining detection/exclusion zones	Incorporates multi-dimensional data, notably DTED, sensor, and (if known) transmitter parameters, allowing highly accurate path loss calculation, essential for non-uniform urban environments; this contrasts with localization methods relying purely on geometric measurements or simplistic path loss assumptions

## Data Availability

The data presented in this study are available on reasonable request from the corresponding author.

## Abbreviations

The following abbreviations are used in this manuscript:

AOA	Angle of Arrival
CF	Coverage Factor
CR	Cognitive Radio
CSS	Cooperative Spectrum Sensing
DOA	Direction Of Arrival
DTED	Digital Terrain Elevation Data
DTM	Digital Terrain Model
ED	Energy Detection
FC	Fusion Center
FDOA	Frequency Difference of Arrival
GNSS	Global Navigation Satellite System
GPS	Global Positioning System
GSM	Global System for Mobile Communications
ISAC	Integrated Sensing and Communication
NR	New Radio
OFDM	Orthogonal Frequency Division Multiplexing
REM	Radio Environment Map
RSS	Received Signal Strength
SDF	Signal Doppler Frequency
SIGINT	Signal Intelligence
SNR	Signal to Noise Ratio
TDOA	Time Difference of Arrival
TOA	Time of Arrival
UAV	Unmanned Aerial Vehicle
UGV	Unmanned Ground Vehicle
UWB	Ultra Wideband
WSN	Wireless Sensor Networks
